# Comorbidity Burden Is Associated with Claims-Based Muscle Wasting and Atrophy Suggestive of Possible Sarcopenia in Korean Adults: A Propensity Score-Matched Analysis Using the National Health Insurance Service Database

**DOI:** 10.3390/healthcare14142072

**Published:** 2026-07-10

**Authors:** Hyunseok Jee, Jimi Kim

**Affiliations:** 1 School of Kinesiology, Yeungnam University, 280 Daehak-ro, Gyeongsan 38541, Gyeongbuk, Republic of Korea; jeehs@ynu.ac.kr; 2 Institute of Sports Science, Yeungnam University, 280 Daehak-ro, Gyeongsan 38541, Gyeongbuk, Republic of Korea; 3 Department of Food and Nutrition, Changwon National University, Changwon 51140, Gyeongnam, Republic of Korea

**Keywords:** possible sarcopenia, comorbidity burden, Charlson comorbidity index, matched case–control study, clinical predictors, ROC analysis

## Abstract

**Background/Objectives**: Possible sarcopenia has been proposed as an early clinical category for identifying individuals at risk of adverse muscle-related outcomes before full diagnostic evaluation. This study examined whether comorbidity burden is associated with possible sarcopenia more strongly than routinely available clinical and laboratory variables. **Methods**: We conducted a retrospective propensity score-matched case–control study using the Korean National Health Insurance Service database (2002–2019). Possible sarcopenia was not defined according to guideline-based muscle strength, muscle mass, or physical performance criteria. Instead, we used KCD code M62.5 as a claims-based proxy for clinically suspected muscle wasting or atrophy suggestive of possible sarcopenia. After exclusion of individuals with missing data, 1793 cases were matched 1:1 with 1793 controls according to age, sex, residential area, and insurance type. Anthropometric measures, biochemical parameters, lifestyle factors, and Charlson Comorbidity Index (CCI) scores were compared. Logistic regression and receiver operating characteristic analyses were performed to evaluate associations and discriminatory performance. **Results**: In the matched population, most anthropometric, biochemical, and lifestyle variables were not significantly different between groups. Fasting blood glucose and gamma-glutamyl transferase were higher in the claims-based possible sarcopenia group, but these associations were not retained in the multivariable model. The CCI score was independently associated with possible sarcopenia (odds ratio: 1.25, 95% confidence interval: 1.20–1.30; *p* < 0.001). In receiver operating characteristic analysis, the CCI showed the highest discriminatory ability among individual predictors (area under the curve, 0.603). The multivariable model yielded an area under the curve of 0.610. **Conclusions**: In administrative health data, claims-recorded muscle wasting or atrophy suggestive of possible sarcopenia was more consistently associated with multimorbidity burden than with individual routine clinical or laboratory markers. These findings support cautious, comorbidity-aware interpretation of muscle wasting/atrophy codes, but do not establish diagnostic validity for possible sarcopenia.

## 1. Introduction

The age-related decline in skeletal muscle health has become a major issue in contemporary clinical practice and public health [1]. Sarcopenia is now understood as a muscle disease characterized by progressive deterioration in muscle strength, muscle mass or quality, and physical performance, and its burden is expected to increase as populations age [1,2]. Because clinically meaningful muscle impairment often develops gradually rather than abruptly, attention has increasingly shifted toward earlier detection [3,4]. In this context, the Asian Working Group for Sarcopenia (AWGS) proposed the term possible sarcopenia to identify individuals who may already be on an adverse trajectory but have not yet undergone full diagnostic evaluation [1]. This concept has particular relevance in community-based and primary care settings, where early screening may enable timely intervention before more severe disability develops [5].

The importance of possible sarcopenia lies in its close relationship with unfavorable health outcomes [1,2]. Reduced muscle strength and sarcopenia have been associated with falls, mobility limitation, frailty, loss of independence, hospitalization, and premature death [6,7]. These findings suggest that early muscle impairment should not be regarded as a benign or inevitable consequence of aging [6,7]. Rather, it may represent a clinically meaningful sign of decreased physiological reserve [8,9]. From a practical perspective, the ability to recognize individuals at higher risk using commonly available healthcare data could improve case finding and support earlier preventive strategies.

Current evidence also indicates that sarcopenia is not an isolated musculoskeletal problem, but part of a broader pattern of multisystem vulnerability [8,10]. Chronic diseases may contribute to muscle impairment through several mechanisms, including persistent inflammation, reduced physical activity, endocrine and metabolic dysregulation, nutritional insufficiency, and vascular compromise [11,12]. At the same time, declining muscle strength may aggravate functional limitation and increase susceptibility to additional disease burden. This bidirectional interplay suggests that the cumulative burden of comorbid conditions may be highly relevant to the development or presence of possible sarcopenia [13,14]. However, despite growing interest in multimorbidity and muscle health, it remains uncertain whether overall comorbidity burden provides more clinically useful information than individual routine clinical or laboratory markers [15].

Another unresolved issue is that much of the existing literature has focused on direct measurements such as handgrip strength, gait speed, nutritional status, or disease-specific cohorts [16]. By comparison, fewer large-scale population-based studies have examined possible sarcopenia using routinely collected healthcare data while simultaneously evaluating anthropometric measures, metabolic indicators, lifestyle factors, and summary indices of comorbidity burden within the same analytic framework [17,18]. This question is important because such variables are widely available in national health screening and administrative databases and may therefore be useful for scalable risk stratification in real-world settings.

Given the recent shift toward broader muscle health frameworks and international harmonization of sarcopenia definitions, this study was not intended to validate a diagnostic definition of sarcopenia. Rather, it aimed to examine whether claims-recorded muscle wasting or atrophy, used as an administrative proxy suggestive of possible sarcopenia, is more strongly associated with multimorbidity than with isolated routine clinical or laboratory markers. This approach may help clarify how claims-recorded muscle wasting/atrophy related to routinely available health-screening variables when direct sarcopenia measurements are unavailable.

Therefore, we conducted a propensity score-matched analysis using the Korean National Health Insurance Service database to compare adults with possible sarcopenia and matched controls and to evaluate whether comorbidity burden may provide more informative risk stratification than individual routine clinical markers.

## 2. Materials and Methods

### 2.1. Study Design and Data Source

This retrospective matched case–control study used data from the National Health Insurance Service (NHIS) database of South Korea from 2002 to 2019. The Korean National Health Insurance is a mandatory single-payer national health insurance system that covers approximately 97% of the Korean population, while the remaining population is covered through the Medical Aid program. Claims data therefore include healthcare utilization from both public and private healthcare providers that are reimbursed through the national insurance system. The NHIS database contains de-identified information on demographic characteristics, eligibility status, health screening results, and healthcare utilization for a nationally representative insured population. Similar population-based analyses using Korean NHIS data have been reported previously [19]. We performed matched comparisons of baseline characteristics, sex-stratified analyses, logistic regression analyses, and receiver operating characteristic (ROC) analyses to identify factors associated with possible sarcopenia and to assess their discriminatory performance.

### 2.2. Study Population

**Figure 1:**
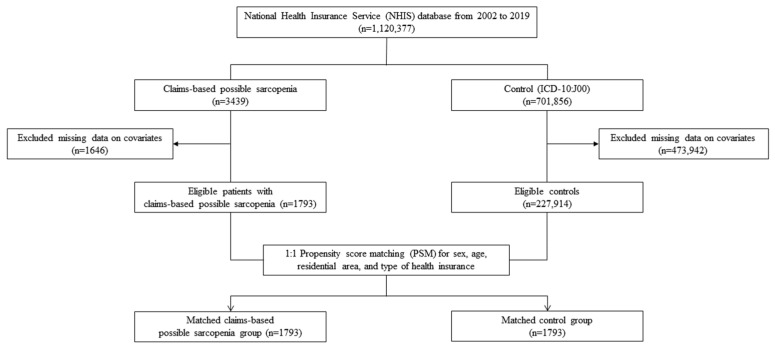
Flow chart of study participant selection and propensity score matching for claims-based possible sarcopenia. Abbreviation: NHIS, National Health Insurance Service; PSM, propensity score matching. Note: Possible sarcopenia was operationally defined using KCD code M62.5 (muscle wasting and atrophy, not elsewhere classified) as a clinically relevant surrogate marker. The control group consisted of patients diagnosed only with acute nasopharyngitis (ICD-10:J00) without other underlying diseases.

The initial source population consisted of 1,120,377 individuals registered in the National Health Insurance Service (NHIS) database between 2002 and 2019. The NHIS cohort included adults aged 20 years or older who participated in the national health screening program during the study period. The mean age of the source population before matching was 50.5 years, whereas the matched analytic population had a mean age of approximately 61 years. As shown in Figure 1, 3439 individuals with claims-based possible sarcopenia and 701,856 potential controls with ICD-10 code J00 were initially identified. As the NHIS data only includes records for individuals seeking medical treatment, potential controls were restricted to those diagnosed solely with acute nasopharyngitis (ICD-10:J00); individuals who received prescriptions for any concurrent conditions were excluded to ensure a true low-severity control group. Acute nasopharyngitis (ICD-10:J00) was selected because it is a common, self-limiting illness that generally reflects transient healthcare utilization without chronic systemic disease. Although other minor acute conditions could also have been considered, J00 provided a large, relatively homogeneous reference population while minimizing underlying multimorbidity. Accordingly, this group was considered an appropriate reference population for comparison in the NHIS database while also allowing the identification of a sufficiently large pool of eligible controls. After excluding participants with missing covariate data, 1793 eligible individuals with claims-based possible sarcopenia and 227,914 eligible controls remained for analysis. To minimize baseline imbalance, 1:1 propensity score matching (PSM) was performed using sex, age, residential area, and type of health insurance. After matching, the final analytic sample consisted of 1793 individuals with claims-based possible sarcopenia and 1793 matched controls. Post-matching balance was assessed using standardized mean differences, and all reported absolute standardized mean differences were below 0.1, indicating acceptable covariate balance. For sex-stratified analyses, the matched male subgroup included 815 controls and 812 individuals with claims-based possible sarcopenia, whereas the matched female subgroup included 978 controls and 981 individuals with claims-based possible sarcopenia.

This study used anonymized secondary data from the NHIS database. Because all participant identifiers were removed before analysis, informed consent was waived. Similar exemptions or waivers have been applied in previous studies using de-identified Korean national health insurance data. This study was conducted in accordance with the Declaration of Helsinki. The study protocol was approved by the Institutional Review Board of Yeungnam University (No. 7002016-E-2024-079).

### 2.3. Definition of Possible Sarcopenia and Control Group

In this study, the outcome was defined as claims-based possible sarcopenia, operationalized using KCD code M62.5 for muscle wasting and atrophy, not elsewhere classified. Because direct measures of handgrip strength, gait speed, chair-stand performance, appendicular skeletal muscle mass, or body composition were not available in the NHIS claims dataset, this definition should be regarded as an administrative proxy rather than a guideline-based diagnostic definition of sarcopenia. Therefore, this outcome may include non-sarcopenic causes of muscle loss, and the findings should be interpreted as claims-based association rather than diagnostic validation.

Although sarcopenia was formally assigned a dedicated disease code in Korea beginning in 2021, clinicians had commonly used M62.5 as closest available diagnostic code for patients with clinically suspected muscle wasting before formal coding became available. The control group consisted of individuals diagnosed only with acute nasopharyngitis (ICD-10:J00). This group was selected to represent a relatively healthy comparison population.

### 2.4. Study Variables

Baseline variables included anthropometric and clinical measurements, biochemical markers, lifestyle factors, and comorbidity burden. Anthropometric and clinical variables were body mass index (BMI, kg/m^2^), waist circumference (cm), systolic blood pressure (mmHg), and diastolic blood pressure (mmHg). Biochemical variables included fasting blood glucose (mg/dL), total cholesterol (mg/dL), triglycerides (mg/dL), high-density lipoprotein cholesterol (HDL-C, mg/dL), low-density lipoprotein cholesterol (LDL-C, mg/dL), hemoglobin (g/dL), serum creatinine (mg/dL), aspartate aminotransferase (AST, IU/L), alanine aminotransferase (ALT, IU/L), and gamma-glutamyl transferase (GGT, IU/L). Proteinuria was categorized as negative, trace, 1+, 2+, 3+, or 4+. These variables correspond to the baseline characteristics and regression variables used in the analysis. Lifestyle factors included smoking status, alcohol consumption, and high- and moderate-intensity physical activity, which were obtained from self-reported health screening questionnaires routinely collected in the NHIS database. Comorbidity burden was assessed using the Charlson Comorbidity Index (CCI) score and further categorized as 0, 1–2, and ≥3. The Charlson Comorbidity Index was calculated using established weighted scores assigned to predefined chronic diseases. Higher scores indicate a greater cumulative burden of comorbidity and are associated with poorer long-term prognoses. For descriptive analyses, CCI scores were categorized as 0 (no recorded comorbidity), 1–2 (low-to-moderate comorbidity burden), and ≥3 (high comorbidity burden). In addition, the prevalences of selected comorbidities, including pulmonary disease, peptic ulcer disease, diabetes mellitus, peripheral vascular disease, and cerebrovascular accident, were summarized.

### 2.5. Statistical Analysis

Continuous variables are presented as mean ± standard deviation, and categorical variables are presented as number (%). Baseline characteristics were compared between the matched control and claims-based possible sarcopenia groups using the *t*-test for continuous variables and the chi-square test for categorical variables. Standardized mean differences were additionally used to evaluate balance before and after matching, with an absolute value < 0.1 considered to indicate acceptable covariate balance. Because the study participants were matched 1:1, conditional logistic regression stratified by matched-pair identifier was used to estimate odds ratios (ORs) and 95% confidence intervals (CIs) for factors associated with possible sarcopenia. Each matched stratum consisted of one participant with possible sarcopenia and one matched control. Crude models included each predictor separately, whereas the multivariable model simultaneously included the prespecified anthropometric, biochemical, lifestyle, and comorbidity variables. In the multivariable model, waist circumference and diastolic blood pressure were excluded because of high correlations with BMI (r = 0.755) and systolic blood pressure (r = 0.682), respectively. Smoking status and physical activity variables were retained as forced covariates regardless of statistical significance because of their clinical relevance to muscle health and metabolic status. Overall fit of the multivariable conditional logistic regression model was assessed using the conditional likelihood-ratio test and Akaike information criterion (AIC). Explanatory performance was summarized using the max-rescaled pseudo-R^2^. Because pseudo-R^2^ statistics in logistic regression are not directly equivalent to the coefficient of determination in ordinary linear regression, the max-rescaled pseudo-R^2^ was interpreted as a relative measure of explanatory performance rather than as a literal percentage of outcome variance explained.

ROC curve analysis was used to evaluate the discriminatory ability of individual predictors for possible sarcopenia. The area under the curve (AUC), optimal cutoff value, sensitivity, and specificity were calculated for each variable, and optimal cutoff values were determined using the Youden index. The discriminatory performance of the multivariable conditional logistic regression model was assessed using the AUC and its 95% CI. All statistical analyses were performed using SAS software (version 9.4; SAS Institute Inc., Cary, NC, USA) and R software (version 4.3.1; R Foundation for Statistical Computing, Vienna, Austria), and a two-sided *p*-value ≤ 0.05 was considered statistically significant.

## 3. Results

### 3.1. Baseline Characteristics Before and After Matching

Before matching, the claims-based possible sarcopenia group was older than the control group (*p* < 0.001), and significant differences were also observed in sex, residential area, and type of health insurance (Supplementary Table S1). After 1:1 matching, however, the two groups were well balanced with respect to age, sex, residential area, and type of health insurance. In addition, all presented covariates showed absolute standardized mean differences below 0.1, indicating adequate matching between the two groups (Supplementary Table S1).

The matched analysis included 1793 controls and 1793 participants with possible sarcopenia, as shown in Table 1. No significant differences were observed between the two groups in BMI, waist circumference, systolic blood pressure, or diastolic blood pressure. Among the biochemical parameters, fasting blood glucose (*p* = 0.016) and GGT (*p* = 0.036) were significantly higher in the claims-based possible sarcopenia group. In contrast, lipid profiles, hemoglobin, serum creatinine, AST, ALT, and proteinuria did not differ significantly between the two groups. Likewise, smoking status, alcohol consumption, and both high- and moderate-intensity physical activity were not significantly different. However, comorbidity burden was substantially greater in the claims-based possible sarcopenia group, with a higher mean CCI score (2.53 ± 2.36 vs. 1.68 ± 1.71, *p* < 0.001). The proportion of participants with a CCI score ≥ 3 was also higher in the claims-based possible sarcopenia group (40.2% vs. 24.3%), and the prevalences of pulmonary disease, peptic ulcer disease, diabetes mellitus, peripheral vascular disease, and cerebrovascular accident were all significantly higher in this group (all *p* < 0.001).

### 3.2. Sex-Stratified Analysis

In the sex-stratified analysis, 815 men in the control group were matched to 812 men with possible sarcopenia, and 978 women in the control group were matched to 981 women with possible sarcopenia (Table 2). Among men, fasting blood glucose was significantly higher in the claims-based possible sarcopenia group than in the control group (*p* = 0.025), whereas hemoglobin was significantly lower (*p* = 0.006). GGT levels were also higher in men with possible sarcopenia (*p* = 0.025). However, no significant differences were observed in BMI, waist circumference, blood pressure, lipid profiles, serum creatinine, AST, ALT, proteinuria, smoking, alcohol consumption, or physical activity. Among men, comorbidity burden remained substantially higher in the claims-based possible sarcopenia group. The mean CCI score was 2.60 ± 2.56 in the claims-based possible sarcopenia group and 1.53 ± 1.70 in the control group (*p* < 0.001), and the proportion with CCI ≥ 3 was 41.0% versus 20.4%, respectively (*p* < 0.001). Pulmonary disease (*p* < 0.001), peptic ulcer disease (*p* < 0.001), diabetes mellitus (*p* < 0.001), peripheral vascular disease (*p* = 0.008), and cerebrovascular accident (*p* < 0.001) were all more prevalent in men with possible sarcopenia. Among women, most clinical and biochemical variables, including fasting blood glucose, hemoglobin, and GGT, did not differ significantly between groups. Nevertheless, comorbidity burden was again significantly higher in women with possible sarcopenia than in controls. The mean CCI score was 2.48 ± 2.17 in the claims-based possible sarcopenia group and 1.80 ± 1.71 in the control group (*p* < 0.001), and the proportion with CCI ≥ 3 was 39.5% versus 27.5%, respectively (*p* < 0.001). Peptic ulcer disease (*p* < 0.001), diabetes mellitus (*p* < 0.001), peripheral vascular disease (*p* = 0.002), and cerebrovascular accident (*p* < 0.001) were more prevalent in women with possible sarcopenia, whereas pulmonary disease did not significantly differ between groups.

### 3.3. Factors Associated with Possible Sarcopenia

In the crude conditional logistic regression analysis, fasting blood glucose (OR = 1.00, 95% CI = 1.00–1.01, *p* = 0.017), GGT (OR = 1.00, 95% CI = 1.00–1.01, *p* = 0.040), and CCI score (OR = 1.23, 95% CI = 1.19–1.28, *p* < 0.001) were significantly associated with the risk of possible sarcopenia (Table 3). In contrast, BMI, waist circumference, blood pressure, lipid parameters, hemoglobin, serum creatinine, AST, ALT, proteinuria, smoking status, and physical activity were not significantly associated with possible sarcopenia in the crude model. In the multivariable conditional logistic regression model, only the CCI score remained independently associated with possible sarcopenia (OR = 1.25, 95% CI = 1.20–1.30, *p* < 0.001). Serum creatinine showed a borderline association. Fasting blood glucose, GGT, BMI, systolic blood pressure, smoking status, and both high- and moderate-intensity physical activity were not significantly associated with possible sarcopenia after adjustment. Waist circumference and diastolic blood pressure were excluded from the multivariable model because of high correlations with BMI and systolic blood pressure, respectively.

### 3.4. ROC Analysis of Individual Predictors

Receiver operating characteristic (ROC) analysis showed that most individual clinical and laboratory variables had limited discriminatory ability for identifying possible sarcopenia (Table 4). The AUC values were 0.498 for BMI, 0.514 for waist circumference, 0.501 for systolic blood pressure, and 0.503 for diastolic blood pressure. Among biochemical markers, the AUC values were 0.516 for fasting blood glucose, 0.508 for total cholesterol, 0.500 for triglycerides, 0.504 for HDL cholesterol, 0.512 for LDL cholesterol, 0.495 for hemoglobin, 0.490 for serum creatinine, 0.507 for AST, 0.511 for ALT, and 0.521 for GGT. The CCI score showed the highest discriminatory performance among the individual predictors, with an AUC of 0.603. The optimal cutoff value for the CCI score was 2.50, corresponding to a sensitivity of 40.2% and a specificity of 75.7%. These findings were consistent with Supplementary Figure S1, in which most individual predictors showed AUC values close to 0.50, whereas the CCI score demonstrated the highest discriminatory ability among the single predictors.

### 3.5. Overall Fit and Discriminatory Performance of the Multivariable Conditional Logistic Regression Model

**Figure 2:**
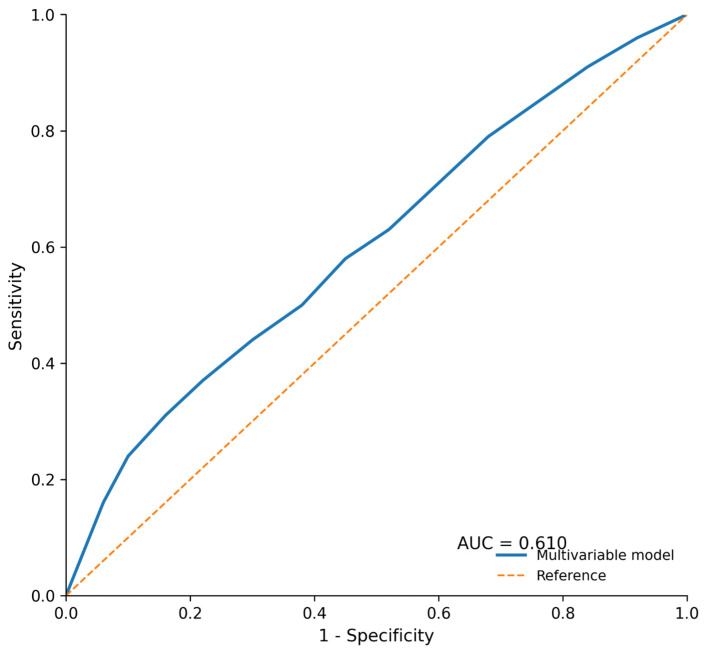
Receiver operating characteristic curve of the multivariable conditional logistic regression model for claims-based possible sarcopenia. The model yielded an area under the curve of 0.610 (95% confidence interval, 0.592–0.627), indicating modest discriminatory performance. The diagonal line represents discrimination equivalent to chance.

The final multivariable conditional logistic regression model provided a statistically significant improvement in fit compared with the conditional null model (likelihood ratio χ^2^ = 158.10, df = 34, *p* < 0.001). The AIC of the final model was 2394.30, and the max-rescaled pseudo-R^2^ was 0.086, indicating limited explanatory performance. The model yielded an AUC of 0.610 (95% CI, 0.592–0.627), indicating modest discriminatory performance for distinguishing participants with possible sarcopenia from their matched controls (Figure 2).

## 4. Discussion

Because direct measurements of muscle strength, muscle mass, and physical performance were unavailable, the present outcome should be interpreted as claims-based possible sarcopenia rather than guideline-confirmed sarcopenia. Accordingly, our findings describe epidemiologic associations within administrative health data and should not be interpreted as diagnostic validation of sarcopenia. Within this operational framework, the matched analysis suggests that claims-based possible sarcopenia is associated more consistently with cumulative comorbidity burden than with isolated abnormalities in routine clinical or laboratory markers.

Within this operational framework, the matched analysis suggests that the clinical profile of possible sarcopenia is driven less by isolated abnormalities in routine biomarkers and more by the accumulated burden of chronic illness. Individuals with claims-based possible sarcopenia consistently showed a greater comorbidity burden than matched controls, and the CCI remained independently associated with possible sarcopenia after multivariable adjustment. We agree that the association between muscle wasting and chronic disease burden is biologically expected. The contribution of this study is therefore not the demonstration that chronic disease are related to muscle deterioration. Rather, the study shows that, within a large claims-based health screening dataset, an aggregate comorbidity measure was more consistently associated with claims-based possible sarcopenia than individual anthropometric, metabolic, biochemical, or lifestyle variables. By contrast, most anthropometric, biochemical, and lifestyle variables did not show persistent independent associations, and their discriminatory performance was limited.

Although fasting glucose and GGT were associated with possible sarcopenia in univariable analyses, these associations disappeared after multivariable adjustment. This attenuation suggests that the observed metabolic differences were largely explained by the broader burden of chronic disease represented by the CCI rather than by independent metabolic effects. Because many chronic conditions represented by the CCI are also associated with altered glucose metabolism and liver enzyme abnormalities, the loss of statistical significance after adjustment likely reflects overlapping disease burden and shared disease pathways rather than independent metabolic effects. In ROC analysis, CCI had the highest performance among the individual predictors, but the magnitude of discrimination was still modest. Taken together, these findings indicate that possible sarcopenia, as captured in this database, may be better understood as part of a broader pattern of multimorbidity than as the consequence of a single dominant metabolic or laboratory abnormality.

This interpretation is compatible with the conceptual background of possible sarcopenia [1]. The AWGS 2019 consensus proposed possible sarcopenia as an early, practical category intended to facilitate case finding before full diagnostic confirmation is feasible [1]. In that sense, possible sarcopenia is not simply a milder version of established sarcopenia; it may also represent an early clinical signal of declining physiologic reserve and increased vulnerability [8,20]. This framework helps explain why the burden of coexisting disease was more informative than single test results in the current analysis [21]. If possible sarcopenia reflects early systemic vulnerability, then a cumulative comorbidity measure may be expected to track it more closely than any one laboratory marker measured at a single time point [22]. The current findings also fit with prior evidence linking muscle weakness and multimorbidity. A recent systematic review found that dynapenia in older adults is associated with a greater likelihood of multimorbidity, supporting the view that reduced muscle strength tends to cluster with chronic disease burden rather than appear in isolation [23]. This broader pattern is important because it implies that possible sarcopenia may emerge through the cumulative effects of multiple chronic disorders, repeated physiologic stress, and progressive declines in reserve capacity [24]. In that context, CCI may function not as a disease-specific marker, but as an integrative summary of systemic health burden [25,26]. That interpretation is supported by the present data, in which the claims-based possible sarcopenia group had markedly higher CCI values and a greater proportion of participants with high comorbidity scores in both sexes [20,27]. The added value of the CCI lies in its ability to summarize cumulative disease burden across multiple organ systems, whereas individual routine markers such as fasting glucose, liver enzymes, creatinine, or hemoglobin capture only isolated physiologic domains at a single time point. Thus, although the CCI showed only modest discrimination, it provided broader clinical context that was not captured by single anthropometric, biochemical, or lifestyle-related variables.

The sex-stratified results add further perspective [28,29]. Among men, fasting glucose and GGT were higher and hemoglobin was lower in the claims-based possible sarcopenia group, whereas among women these biochemical differences were less pronounced after matching. However, the association between possible sarcopenia and comorbidity burden remained directionally similar in both sexes. This pattern suggests that sex-specific biologic signals may exist, but that the broader link between possible sarcopenia and disease burden is more stable than any individual marker profile [2,8]. In other words, although certain laboratory differences may emerge in one sex more than the other, they do not appear to outweigh the common underlying pattern of greater multimorbidity [30]. Another noteworthy result is the weak discriminatory ability of most individual markers [31]. Even though CCI performed better than the other single predictors, its AUC remained only 0.603, and the multivariable model reached 0.610. These values do not support the use of CCI or the current laboratory panel as a standalone screening substitute for direct sarcopenia-related assessment. Rather, they suggest a narrower but still useful role: identifying a subgroup of individuals in whom the probability of possible sarcopenia may be higher and who may therefore benefit from additional evaluation [32]. This distinction matters. The data do not justify claiming that CCI is a strong predictor in an absolute sense; instead, CCI appears to be the most informative single correlate among the variables available in this dataset [33].

A biologically plausible explanation for these findings is that possible sarcopenia reflects the convergence of multiple pathophysiologic processes [34,35]. Chronic inflammation, inactivity, metabolic dysregulation, oxidative stress, endocrine imbalance, vascular insufficiency, and nutritional deterioration can all impair muscle quality and strength [35,36,37]. Because these mechanisms often coexist across chronic diseases, a summary measure of comorbidity may indirectly capture their cumulative effects better than isolated biomarkers [30]. This may explain why single measurements such as fasting glucose, hemoglobin, creatinine, or liver enzymes performed poorly in ROC analysis [31]. Each marker reflects only one fragment of a much larger biologic process, whereas multimorbidity may better represent the long-term systemic context in which muscle dysfunction develops [38]. Some of the disease-specific patterns observed in this study are also clinically coherent. The higher prevalence of diabetes mellitus in the claims-based possible sarcopenia group is consistent with the well-established interaction between impaired glucose metabolism and skeletal muscle dysfunction [39,40]. Skeletal muscle is central to insulin-mediated glucose disposal, and insulin resistance may reduce anabolic signaling and impair muscle quality [41,42]. At the same time, poor muscle function may worsen metabolic regulation, creating a reinforcing cycle [43]. In the present analysis, however, fasting glucose did not remain independently associated after adjustment, which suggests that glucose dysregulation alone may not explain the observed phenotype once broader comorbidity burden is considered. The higher prevalence of pulmonary disease is similarly plausible. Chronic respiratory disease is frequently accompanied by inactivity, systemic inflammation, hypoxemia, nutritional depletion, oxidative stress, and corticosteroid exposure, all of which may contribute to muscle decline [44,45]. Meta-analytic evidence has shown that sarcopenia is common in COPD and is associated with poorer clinical outcomes [46]. Our results do not establish a causal pathway, but they do support the idea that pulmonary disease forms part of the larger multimorbidity pattern associated with possible sarcopenia. The observed excess of cerebrovascular accident and peripheral vascular disease in the claims-based possible sarcopenia group may also reflect plausible biological links. Stroke can accelerate functional decline through immobilization, impaired motor control, swallowing difficulty, and catabolic stress, while peripheral vascular disease may reduce tissue perfusion, limit physical activity, and coexist with broader cardiometabolic abnormalities [47,48,49]. These disorders may therefore act as markers of both direct physiologic burden and accumulated functional compromise [8]. Likewise, the higher GGT levels observed particularly in men may indicate oxidative or metabolic stress, but the lack of an independent association after adjustment suggests that GGT should be interpreted cautiously as a nonspecific correlate rather than a reliable standalone indicator of possible sarcopenia. The association with peptic ulcer disease is less immediately intuitive, but it is not without precedent [50]. A nationwide population-based study previously reported an independent association between sarcopenia and peptic ulcer disease [51]. One reasonable interpretation is that peptic ulcer disease may reflect a broader profile of frailty, nutritional vulnerability, medication exposure, or chronic illness rather than a direct ulcer-to-muscle pathway [52,53,54]. Our findings are compatible with that view and suggest that peptic ulcer disease should be considered as part of the wider multimorbidity landscape rather than as an isolated mechanistic driver.

From a clinical perspective, these results suggest that comorbidity-aware interpretation may be useful when possible sarcopenia is evaluated using routine healthcare or administrative data. In situations where direct measures of muscle strength or physical performance are unavailable, a high burden of chronic disease may help identify individuals who warrant closer attention [55,56]. At the same time, the modest AUC values observed here make it clear that multimorbidity cannot replace direct screening tools. The more realistic implication is that comorbidity burden may enrich pretest suspicion and support prioritization for confirmatory assessment, rather than functioning as a diagnostic endpoint in itself. This interpretation is consistent with the practical intent of the AWGS possible sarcopenia construct [57].

This study has several strengths. It drew on a large national database and included a broad source population with routinely collected demographic, clinical, laboratory, lifestyle, and claims-based information. Propensity score matching improved comparability between groups on key background characteristics, and sex-stratified analyses allowed us to examine whether the observed patterns were consistent across men and women. In addition, the study assessed both association and discrimination, which provides a more clinically informative picture than reporting odds ratios alone. Several limitations should be considered when interpreting the results. First, possible sarcopenia was operationally defined using KCD code M62.5 because no specific diagnostic code for sarcopenia existed during most of the study period. Although this coding approach reflects historical clinical practice in Korea, it has not been formally validated against direct assessments of muscle strength, muscle mass, or physical performance. In addition, the use of KCD code M62.5 may have varied across clinicians and institutions depending on clinical suspicion, documentation practices, and coding behavior, which could have introduced additional outcome misclassification. Therefore, some degree of outcome misclassification cannot be excluded. Furthermore, the present claims-based operational definition has not been externally validated in independent dataset or against standardized sarcopenia assessment. Second, the study design was retrospective and observational, so temporal direction and causality cannot be established. Third, residual confounding remains possible because detailed data on diet, medication use, inflammatory biomarkers, disease severity, and direct functional status were not available. Fourth, because the control group consisted of individuals with only acute nasopharyngitis (J00) and without concurrent prescriptions, the observed association should be interpreted as reflecting differences between individuals with relatively high multimorbidity and those with very low disease burden. Therefore, the magnitude of the observed association may not be directly generalizable to the broader community population with heterogeneous health conditions. Nevertheless, previous epidemiological studies have consistently demonstrated an association between multimorbidity and muscle impairment, suggesting that the direction of the observed association may remain similar in more representative populations, although its magnitude could differ.

In summary, this matched analysis indicates that possible sarcopenia is more closely aligned with cumulative comorbidity burden than with most single routine clinical or biochemical markers. Among the evaluated variables, the CCI emerged as the most informative individual predictors, although its overall discriminatory ability remained modest. Although the discriminatory performance was modest, the CCI may provide complementary information for identifying individuals who may benefit from further clinical evaluation rather than functioning as a stand-alone screening tool. These findings support interpreting possible sarcopenia within a multimorbidity framework and suggest that comorbidity burden may help identify individuals who warrant more direct assessment of muscle-related function. Future studies should validate these observations using standardized sarcopenia-related measurements, refine claims-based operational definitions, and determine whether combining comorbidity indices with direct functional screening improves early identification in routine practice.

## 5. Conclusions

In conclusion, this propensity score-matched analysis using the Korean NHIS database showed that claims-recorded muscle wasting or atrophy suggestive of possible sarcopenia was consistently associated with cumulative comorbidity burden than with individual anthropometric, biochemical, or lifestyle-related variables. The CCI was the only factor that remained independently associated with this claims-based outcome and demonstrated the highest, although modest, discriminatory performance among the evaluated predictors. These findings suggest that claims-recorded muscle wasting or atrophy may reflect a broader state of multimorbidity and reduced physiological reserve rather than a condition explained by a single routine clinical marker. However, given the limited discriminatory accuracy, the CCI should not be used as a standalone screening tool for sarcopenia or possible sarcopenia. At most, it may provide ancillary information for identifying individuals with claims-recorded muscle wasting or atrophy who may warrant objective assessment of muscle strength, muscle mass, and physical function. These findings should therefore be interpreted as administrative epidemiological signals rather than evidence validating a diagnostic definition of possible sarcopenia. Future studies incorporating standardized functional and body composition measurements are needed to validate claims-based definitions and to develop more accurate identification strategies that combine comorbidity information with direct muscle-related assessments.

## Figures and Tables

**Table 1:** Baseline characteristics of the matched study population.

Variables	Matched Control (*n* = 1793)	Matched Claims-Based Possible Sarcopenia (*n* = 1793)	*p*-Value
Anthropometric and clinical measurements			
BMI (kg/m^2^)	24.07 ± 3.21	24.05 ± 3.39	0.91
Waist circumference (cm)	81.53 ± 8.99	82.01 ± 9.29	0.12
Systolic blood pressure (mmHg)	126.18 ± 16.21	126.16 ± 15.97	0.97
Diastolic blood pressure (mmHg)	77.20 ± 10.09	77.19 ± 10.03	0.98
Biochemical measurements			
Fasting blood glucose (mg/dL)	102.09 ± 27.36	104.47 ± 31.49	0.016
Total cholesterol (mg/dL)	195.52 ± 39.56	194.62 ± 39.64	0.50
Triglycerides (mg/dL)	135.62 ± 124.54	136.19 ± 95.08	0.88
HDL cholesterol (mg/dL)	52.04 ± 19.03	54.04 ± 14.06	0.99
LDL cholesterol (mg/dL)	116.79 ± 56.41	114.05 ± 39.74	0.09
Hemoglobin (g/dL)	13.66 ± 1.65	13.62 ± 1.58	0.50
Serum creatinine (mg/dL)	1.02 ± 1.09	0.94 ± 0.72	0.12
AST (IU/L)	27.07 ± 39.12	28.28 ± 35.57	0.34
ALT (IU/L)	24.70 ± 27.46	25.31 ± 23.49	0.48
GGT (IU/L)	37.18 ± 54.10	41.84 ± 76.81	0.036
Proteinuria (%) *			
Negative (−)	1694 (94.5)	1678 (93.6)	0.35
Trace (±)	39 (2.2)	45 (2.5)	
1+	38 (2.1)	39 (2.2)	
2+	15 (0.8)	23 (1.3)	
3+	3 (0.2)	7 (0.4)	
4+	4 (0.2)	1 (0.1)	
Lifestyle factors			
Smoking status (%)			
Never	1224 (68.3)	1229 (68.5)	0.98
Former	266 (14.8)	262 (14.6)	
Current	303 (16.9)	302 (16.8)	
Alcohol consumption (days/week)	0.81 ± 1.48	0.80 ± 1.51	0.97
High-intensity physical activity (days/week)	0.91 ± 1.70	0.84 ± 1.62	0.19
Moderate-intensity physical activity (days/week)	1.21 ± 1.95	1.12 ± 1.90	0.16
Comorbidities			
Charlson Comorbidity Index (CCI) score	1.68 ± 1.71	2.53 ± 2.36	<0.001
CCI category (%)			
0	506 (28.2)	347 (19.4)	<0.001
1–2	852 (47.5)	726 (40.5)	
≥3	435 (24.3)	720 (40.2)	
Prevalent comorbidities (%) †			
Pulmonary disease	702 (39.2)	835 (46.6)	<0.001
Peptic ulcer	637 (35.5)	814 (45.4)	<0.001
Diabetes mellitus	375 (20.9)	564 (31.5)	<0.001
Peripheral vascular disease	241 (13.4)	331 (18.5)	<0.001
Cerebrovascular accident	202 (11.3)	382 (21.3)	<0.001

ALT, alanine aminotransferase; AST, aspartate aminotransferase; CCI, Charlson Comorbidity Index; GGT, gamma-glutamyl transferase; HDL, high-density lipoprotein; LDL, low-density lipoprotein. Data are presented as mean ± SD for continuous variables and number (%) for categorical variables. *p*-values were determined using *t*-test for continuous variables and the Chi-square test for categorical variables. Age, sex, residential area, and type of health insurance were used as 1:1 propensity score matching variables and were therefore omitted from this table. * Urine protein was measured using a dipstick test and categorized into six levels. † Only the five most prevalent comorbidities are listed.

**Table 2:** Baseline characteristics of the matched study population stratified by sex.

	Men		*p*-Value	Women		*p*-Value
Variables	Matched Control (*n* = 815)	Matched Claims-Based Possible Sarcopenia (*n* = 812)		Matched Control Control (*n* = 978)	Matched Claims-Based Possible Sarcopenia (*n* = 981)	
Anthropometric and clinical measurements						
BMI (kg/m^2^)	24.09 ± 3.01	24.13 ± 3.32	0.82	24.05 ± 3.38	23.99 ± 3.44	0.73
Waist circumference (cm)	84.24 ± 8.29	84.97 ± 8.76	0.08	79.27 ± 8.92	79.56 ± 9.00	0.48
Systolic blood pressure (mmHg)	127.27 ± 15.43	127.48 ± 15.34	0.78	125.27 ± 16.78	125.06 ± 16.41	0.78
Diastolic blood pressure (mmHg)	78.12 ± 9.94	78.73 ± 10.03	0.21	76.43 ± 10.17	75.92 ± 9.85	0.26
Biochemical measurements						
Fasting blood glucose (mg/dL)	104.46 ± 30.76	108.15 ± 35.32	0.025	100.13 ± 24.00	101.43 ± 27.58	0.27
Total cholesterol (mg/dL)	191.38 ± 36.04	189.79 ± 39.12	0.39	198.96 ± 41.97	198.61 ± 39.65	0.85
Triglycerides (mg/dL)	148.19 ± 165.82	141.49 ± 92.29	0.31	125.14 ± 72.80	131.80 ± 97.15	0.09
HDL cholesterol (mg/dL)	51.38 ± 17.00	51.34 ± 13.16	0.96	56.26 ± 20.32	56.27 ± 14.39	0.99
LDL cholesterol (mg/dL)	115.28 ± 71.33	110.44 ± 34.60	0.08	118.05 ± 39.93	117.04 ± 43.33	0.59
Hemoglobin (g/dL)	14.74 ± 1.41	14.55 ± 1.48	0.006	12.75 ± 1.24	12.86 ± 1.21	0.07
Serum creatinine (mg/dL)	1.20 ± 2.65	1.07 ± 0.80	0.20	0.86 ± 0.83	0.83 ± 0.62	0.31
AST (IU/L)	30.26 ± 56.03	31.92 ± 49.72	0.53	24.42 ± 13.27	25.26 ± 15.71	0.20
ALT (IU/L)	28.55 ± 33.12	29.79 ± 25.94	0.40	21.50 ± 21.13	21.60 ± 20.53	0.92
GGT (IU/L)	50.28 ± 68.48	60.28 ± 106.94	0.025	26.27 ± 34.62	26.57 ± 28.45	0.84
Proteinuria (%) *						
Negative (−)	775 (95.1)	758 (93.4)	0.53	919 (94.0)	920 (93.8)	0.54
Trace (±)	15 (1.8)	20 (2.5)		24 (2.5)	25 (2.6)	
1+	16 (2.0)	16 (2.0)		22 (2.3)	23 (2.3)	
2+	6 (0.7)	13 (1.6)		9 (0.9)	10 (1.0)	
3+	2 (0.3)	4 (0.5)		1 (0.1)	3 (0.3)	
4+	1 (0.1)	1 (0.1)		3 (0.3)	0 (0.0)	
Lifestyle factors						
Smoking status (%)						
Never	292 (35.8)	295 (36.3)	0.87	932 (95.3)	934 (95.2)	0.08
Former	250 (30.7)	255 (31.4)		16 (1.6)	7 (0.7)	
Current	273 (33.5)	262 (32.3)		30 (3.1)	40 (4.1)	
Alcohol consumption (days/week)	1.45 ± 1.86	1.41 ± 1.87	0.65	0.30 ± 0.83	0.27 ± 0.70	0.30
High-intensity physical activity (days/week)	1.11 ± 1.78	0.97 ± 1.70	0.12	0.75 ± 1.62	0.73 ± 1.54	0.78
Moderate-intensity physical activity (days/week)	1.38 ± 1.99	1.24 ± 1.97	0.16	1.07 ± 1.90	1.02 ± 1.83	0.54
Comorbidities						
Charlson Comorbidity Index (CCI) score	1.53 ± 1.70	2.60 ± 2.56	<0.001	1.80 ± 1.71	2.48 ± 2.17	<0.001
CCI category (%)						
0	263 (32.3)	188 (23.2)	<0.001	243 (24.9)	159 (16.2)	<0.001
1–2	386 (47.4)	291 (35.8)		466 (47.7)	435 (44.3)	
≥3	166 (20.4)	333 (41.0)		269 (27.5)	387 (39.5)	
Prevalent comorbidities (%) †						
Pulmonary disease	291 (35.7)	385 (47.4)	<0.001	411 (42.0)	450 (45.9)	0.09
Peptic ulcer	251 (30.8)	338 (41.6)	<0.001	386 (39.5)	476 (48.5)	<0.001
Diabetes mellitus	175 (21.5)	267 (32.9)	<0.001	200 (20.5)	297 (30.3)	<0.001
Peripheral vascular disease	99 (12.2)	136 (16.8)	0.008	142 (14.5)	195 (20.0)	0.002
Cerebrovascular accident	80 (9.8)	190 (23.4)	<0.001	122 (12.5)	192 (19.6)	<0.001

ALT, alanine aminotransferase; AST, aspartate aminotransferase; CCI, Charlson Comorbidity Index; GGT, gamma-glutamyl transferase; HDL, high-density lipoprotein; LDL, low-density lipoprotein. Data are presented as mean ± SD for continuous variables and number (%) for categorical variables. *p*-values were determined using *t*-test for continuous variables and the Chi-square test for categorical variables. Age, residential area, and type of health insurance were matching variables and were therefore not included in this table. * Urine protein was measured using a dipstick test and categorized into six levels. † Only the five most prevalent comorbidities are listed.

**Table 3:** Associations of baseline characteristics with the risk of claims-based possible sarcopenia.

	Crude Model		Multivariable Model	
Variables	OR (95% CI)	*p*-Value	OR (95% CI)	*p*-Value
Anthropometric and clinical measurements				
BMI (kg/m^2^)	0.99 (0.98–1.02)	0.91	1.00 (0.98–1.02)	0.77
Waist circumference (cm)	1.01 (1.00–1.01)	0.12	-	-
Systolic blood pressure (mmHg)	1.00 (0.99–1.01)	0.97	1.00 (0.99–1.00)	0.09
Diastolic blood pressure (mmHg)	1.00 (0.99–1.01)	0.99	-	-
Biochemical measurements				
Fasting blood glucose (mg/dL)	1.00 (1.00–1.01)	0.017	1.00 (0.99–1.01)	0.50
Total cholesterol (mg/dL)	1.00 (0.99–1.00)	0.50	1.00 (0.99–1.01)	0.20
Triglycerides (mg/dL)	1.00 (0.99–1.00)	0.88	1.00 (0.99–1.00)	0.91
HDL cholesterol (mg/dL)	1.00 (0.99–1.00)	0.98	1.00 (0.99–1.01)	0.54
LDL cholesterol (mg/dL)	0.99 (0.99–1.00)	0.10	0.99 (0.99–1.00)	0.20
Hemoglobin (g/dL)	0.98 (0.95–1.03)	0.50	1.04 (0.99–1.09)	0.10
Serum creatinine (mg/dL)	0.95 (0.88–1.02)	0.15	0.92 (0.84–1.00)	0.05
AST (IU/L)	1.00 (0.99–1.00)	0.36	1.00 (0.99–1.00)	0.70
ALT (IU/L)	1.00 (0.99–1.00)	0.48	1.00 (0.99–1.00)	0.62
GGT (IU/L)	1.00 (1.00–1.01)	0.040	1.00 (0.99–1.00)	0.34
Proteinuria				
Negative (−)	1.0 (ref)		1.0 (ref)	
Trace (±)	3.96 (0.44–35.49)	0.22	2.81 (0.30–26.30)	0.37
+1	4.62 (0.50–43.04)	0.18	2.95 (0.30–28.79)	0.35
+2	4.11 (0.44–38.42)	0.22	2.68 (0.27–26.20)	0.40
+3	6.13 (0.62–60.31)	0.12	3.53 (0.34–36.32)	0.29
+4	9.33 (0.71–122.57)	0.09	4.26 (0.31–59.34)	0.28
Lifestyle factors				
Smoking status				
Never	1.0 (ref)		1.0 (ref)	
Former	0.98 (0.81–1.18)	0.84	0.95 (0.77–1.16)	0.59
Current	0.99 (0.83–1.19)	0.94	1.03 (0.85–1.26)	0.75
High-intensity physical activity (days/week)				
0	1.0 (ref)		1.0 (ref)	
1	1.08 (0.86–1.36)	0.51	1.18 (0.90–1.55)	0.24
2	0.86 (0.67–1.11)	0.25	0.93 (0.68–1.25)	0.61
3	0.93 (0.70–1.23)	0.60	1.00 (0.72–1.40)	0.98
4	0.83 (0.54–1.29)	0.41	0.93 (0.58–1.50)	0.76
5	1.07 (0.70–1.64)	0.74	1.16 (0.71–1.89)	0.56
6	0.80 (0.45–1.41)	0.44	0.85 (0.41–1.76)	0.65
7	0.81 (0.52–1.26)	0.34	0.82 (0.48–1.38)	0.45
Moderate-intensity physical activity (days/week)				
0	1.0 (ref)		1.0 (ref)	
1	0.94 (0.75–1.18)	0.62	0.87 (0.66–1.14)	0.31
2	0.89 (0.69–1.14)	0.35	0.91 (0.67–1.22)	0.51
3	0.89 (0.69–1.15)	0.37	0.89 (0.66–1.20)	0.45
4	0.81 (0.56–1.16)	0.24	0.82 (0.55–1.22)	0.33
5	0.95 (0.66–1.35)	0.76	0.90 (0.59–1.36)	0.60
6	0.84 (0.51–1.40)	0.51	0.92 (0.48–1.77)	0.81
7	0.90 (0.64–1.26)	0.52	0.98 (0.65–1.46)	0.91
Comorbidities				
Charlson Comorbidity Index (CCI) Score	1.23 (1.19–1.28)	<0.001	1.25 (1.20–1.30)	<0.001

ALT, alanine aminotransferase; AST, aspartate aminotransferase; CCI, Charlson Comorbidity Index; GGT, gamma-glutamyl transferase; HDL, high-density lipoprotein; LDL, low-density lipoprotein. The multivariable-adjusted model (Adjusted Model) evaluated the independent associations between each variable and the risk of possible sarcopenia. Crude and multivariable odds ratios and 95% confidence intervals were estimated using conditional logistic regression stratified by matched-pair identifier. Each matched stratum consisted of one participant with possible sarcopenia and one matched control. Waist circumference and diastolic blood pressure were excluded from the multivariable model to ensure statistical stability, as they exhibited high Pearson correlation coefficients with body mass index (r = 0.755) and systolic blood pressure (r = 0.682), respectively. Lifestyle factors, including smoking status and physical activity (high-intensity and moderate-intensity), were retained as forced covariates in the multivariable model regardless of their statistical significance (*p* > 0.05), given their established clinical and biological relevance to muscle mass and metabolic health. *p*-values < 0.05 were considered statistically significant.

**Table 4:** Receiver operating characteristic analysis of individual predictors for claims-based possible sarcopenia.

Variable	AUC	Optimal Cutoff	Sensitivity (%)	Specificity (%)
Anthropometric and clinical measurements				
BMI (kg/m^2^)	0.498	25.55	31.5	69.7
Waist circumference (cm)	0.514	81.40	52.0	51.1
Systolic blood pressure (mmHg)	0.501	121.50	56.1	45.4
Diastolic blood pressure (mmHg)	0.503	70.50	68.9	33.2
Biochemical measurements				
Fasting blood glucose (mg/dL)	0.516	108.50	26.8	77.5
Total cholesterol (mg/dL)	0.508	196.50	55.3	47.6
Triglycerides (mg/dL)	0.500	99.50	60.6	40.7
HDL cholesterol (mg/dL)	0.504	39.50	87.7	14.9
LDL cholesterol (mg/dL)	0.512	104.50	41.8	60.8
Hemoglobin (g/dL)	0.495	12.75	72.4	28.9
Serum creatinine (mg/dL)	0.490	1.15	12.9	89.6
AST (IU/L)	0.507	24.50	45.9	57.0
ALT (IU/L)	0.511	18.50	57.9	44.4
GGT (IU/L)	0.521	17.50	71.1	33.1
Comorbidities				
Charlson Comorbidity Index (CCI) score	0.603	2.50	40.2	75.7

AUC, area under the curve; BMI, body mass index; HDL, high-density lipoprotein; LDL, low-density lipoprotein; AST, aspartate aminotransferase; ALT, alanine aminotransferase; GGT, gamma-glutamyl transferase; CCI, Charlson Comorbidity Index. Optimal cutoff values were determined using the Youden index. Sensitivity and specificity were calculated at the optimal cutoff point.

## Data Availability

Restrictions apply to the availability of these data. The data used in this study were obtained from the Korean National Health Insurance Service (NHIS) database and are not publicly available due to data protection and privacy regulations. Data may be available from the NHIS Big Data Platform for eligible researchers upon reasonable request and with permission from the NHIS.

## Supplementary Materials

The following supporting information can be downloaded at: https://www.mdpi.com/article/10.3390/healthcare14142072/s1, Table S1. Baseline characteristics of the control and claims-based possible sarcopenia groups before and after matching; Figure S1. Receiver operating characteristic curves of selected individual predictors for possible sarcopenia, including the Charlson Comorbidity Index (CCI) score, waist circumference, systolic blood pressure, total cholesterol, AST, ALT, and LDL cholesterol. Most individual predictors showed limited discriminatory ability, with AUC values close to 0.50, whereas the CCI score showed the highest discriminatory performance among the individual predictors.
