# Supplementary material for: Comorbidity Burden Is Associated with Claims-Based Muscle Wasting and Atrophy Suggestive of Possible Sarcopenia in Korean Adults: A Propensity Score-Matched Analysis Using the National Health Insurance Service Database
Source: Healthcare (Basel). 2026 Jul 10;14(14):2072. doi: 10.3390/healthcare14142072 (PMC13410032; doi:10.3390/healthcare14142072)
Supplement: Supplementary file 1 [file healthcare-14-02072-s001.zip › healthcare-4347246-supplementary.pdf]

Supplementary Table S1. Baseline characteristics of the control and claims-based possible sarcopenia groups before and after matching**.**

Variable	Pre-matching						Post-matching					
	Control (*n* = 227,914)		Claims-based possible sarcopenia (*n* = 1,793)		*P*-value	|SMD|	Control (*n* = 1,793)		Claims-based possible sarcopenia (*n* = 1,793)		*P*-value	|SMD|
	n or mean	% or std	*n* or mean	% or std			*n* or mean	% or std	*n* or mean	% or std		
Age (years)	50.46	14.57	61.49	14.71	<0.001	0.753	61.33	14.5	61.49	14.71	0.74	0.011
Sex, n (%)												
Male	108,577	47.64	812	45.29	0.047	0.047	815	45.45	812	45.29	0.92	0.003
Female	119,337	52.36	981	54.71		0.047	978	54.55	981	54.71		0.003
Residential area, n (%)												
Seoul	42,720	18.74	142	7.92	<0.001	0.322	140	7.81	142	7.92	1.00	0.004
Busan	16,355	7.18	225	12.55		0.181	227	12.66	225	12.55		0.003
Daegu	11,193	4.91	32	1.78		0.175	32	1.78	32	1.78		0.000
Incheon	12,391	5.44	128	7.14		0.070	124	6.92	128	7.14		0.009
Gwangju	6,396	2.81	48	2.68		0.008	46	2.57	48	2.68		0.007
Daejeon	6,812	2.99	42	2.34		0.040	40	2.23	42	2.34		0.007
Ulsan	5,442	2.39	56	3.12		0.045	51	2.84	56	3.12		0.016
Sejong	508	0.22	3	0.17		0.011	2	0.11	3	0.17		0.016
Gyeonggi	52,824	23.18	211	11.77		0.304	210	11.71	211	11.77		0.002
Gangwon	6,718	2.95	243	13.55		0.393	252	14.05	243	13.55		0.014
Chungbuk	7,740	3.4	85	4.74		0.068	82	4.57	85	4.74		0.008
Chungnam	10,629	4.66	116	6.47		0.079	121	6.75	116	6.47		0.011
Jeonbuk	8,919	3.91	105	5.86		0.091	108	6.02	105	5.86		0.007
Jeonnam	9,048	3.97	102	5.69		0.080	104	5.8	102	5.69		0.005
Gyeongbuk	12,852	5.64	108	6.02		0.016	109	6.08	108	6.02		0.003
Gyeongnam	15,129	6.64	123	6.86		0.009	120	6.69	123	6.86		0.007
Jeju	2,238	0.98	24	1.34		0.034	25	1.39	24	1.34		0.004
Type of health insurance, n (%)												
Medicaid & NHI self-employed/ employee subscriber Low	35,092	15.4	318	17.74	<0.001	0.063	320	17.85	318	17.74	1.00	0.003
NHI self-employed subscriber Medium	24,331	10.68	232	12.94			227	12.66	232	12.94		0.008
NHI self-employed subscriber	24,571	10.78	191	10.65			187	10.43	191	10.65		0.007
NHI employee subscriber Medium	79,049	34.68	500	27.89			507	28.28	500	27.89		0.009
NHI employee subscriber High	64,871	28.46	552	30.79			552	30.79	552	30.79		0.000

NHI, National Health Insurance; SMD, Standardized Mean Difference. Data are presented as mean (standard deviation, std) for continuous variables and as number (percentage, %) for categorical variables. An absolute SMD (|SMD|) of < 0.1 indicates an appropriate balance of baseline characteristics between the two groups after matching. The *P*-value indicates the statistical significance of differences between the control and possible sarcopenia groups before and after matching, generally calculated using independent t-tests for continuous variables and Chi-square tests for categorical variables.

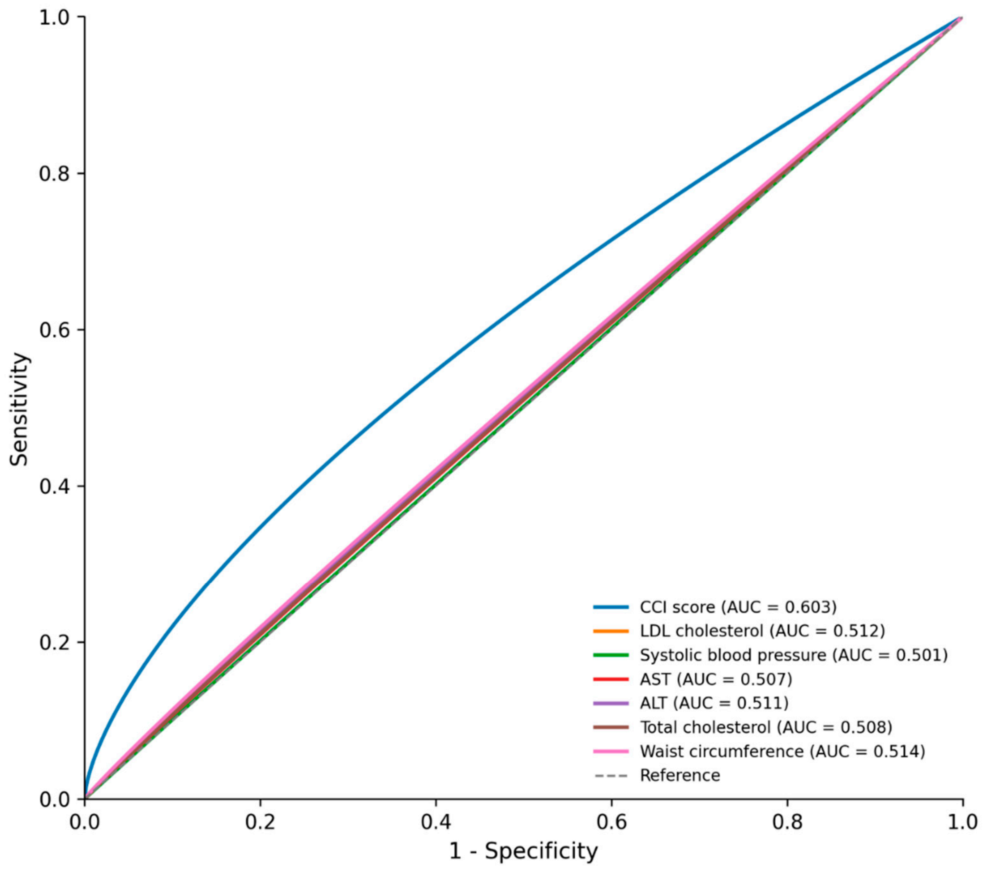

Supplementary Figure S1. Receiver operating characteristic curves of selected individual predictors for possible sarcopenia, including the Charlson Comorbidity Index (CCI) score, waist circumference, systolic blood pressure, total cholesterol, AST, ALT, and LDL cholesterol. Most individual predictors showed limited discriminatory ability, with AUC values close to 0.50, whereas the CCI score showed the highest discriminatory performance among the individual predictors.
